# In *vivo* application of potent probiotics for enhancing potato growth and controlling *Ralstonia solanacearum* and *Fusarium oxysporum* infections

**DOI:** 10.1007/s10482-024-01928-2

**Published:** 2024-02-09

**Authors:** Ahmed Hamdy Abdel-Moghies, Motaz Hassan El-Sehrawy, Abeer Emam Zakaria, Shimaa Mohamed Fahmy

**Affiliations:** 1 Radiation Microbiology Department, National Center for Research and Radiation Technology, Egyptian Atomic Energy Authority, Cairo, Egypt; 2 Botany and Microbiology Department, Faculty of Science, Al-Azhar University, Cairo, Egypt

**Keywords:** Probiotics, *Ralstonia solanacearum*, *Fusarium oxysporum*, Potato cultivation, Radiation response

## Abstract

Plant probiotics are live microbial cells or cultures that support plant growth and control plant pathogens through different mechanisms. They have various effects on plants, including plant growth promotion through the production of indole acetic acid (IAA), biological control activity (BCA), and production of cellulase enzymes, thus inducing systemic resistance and increasing the availability of mineral elements. The present work aimed to study the potential of *Achromobacter marplatensis* and *Bacillus velezensis* as plant probiotics for the field cultivation of potatoes. In *vitro* studies have demonstrated the ability of selected probiotics to produce IAA and cellulase, as well as antimicrobial activity against two plant pathogens that infect *Solanum tuberosum* as *Fusarium oxysporum* and *Ralstonia solanacearum* under different conditions at a broad range of different temperatures and pH values. In *vivo* study of the effects of the probiotics *A. marplatensis* and *B. velezensis* on *S. tuberosum* plants grown in sandy clay loamy soil was detected after cultivation for 90 days. Probiotic isolates *A. marplatensis* and *B. velezensis* were able to tolerate ultraviolet radiation (UV) exposure for up to two hours, the dose response curve exhibited that the D_10_ values of *A. marplatensis* and *B. velezensis* were 28 and 16 respectively. In the case of loading both probiotics with broth, the shoot dry weight was increased significantly from 28 in the control to 50 g, shoot length increased from 24 to 45.7 cm, branches numbers increased from 40 to 70 branch, leaves number increased from 99 to 130 leaf, root dry weight increased from 9.3 to 12.9 g, root length increased from 24 to 35.7 cm, tuber weight increased from 15 to 37.0 g and tubers number increased from 9 to 24.4 tuber, the rot percentage was reduced to 0%. The addition of both probiotic isolates, either broth or wheat grains load separately has enhanced all the growth parameters; however, better results and increased production were in favor of adding probiotics with broth more than wheat. On the other hand, both probiotics showed a remarkable protective effect against potato pathogens separately and reduced the negative impact of the infection using them together.

**Supplementary Information:**

The online version contains supplementary material available at 10.1007/s10482-024-01928-2.

## Introduction

Endophytes are an important group of microorganisms having an intimate relationship with their hosts which mostly colonize intercellular spaces and form a range of different associations with them commensals, symbionts, trophobiotic and mutualists. They boost the growth and development of the plants and also a warehouse of secondary metabolites. Important mechanisms such as biofertilization and phytostimulation involved in growth promotion and biocontrol activity against disease suppression have been reported. There is increasing scope to use these endophytes for disease suppression and plant growth promotion (Krishnappa et al. 2023). Endophytes are planted colonizing microorganisms in a mutualistic symbiosis relationship for plant growth by stimulating the production of plant hormones, such as auxins, or as plant protective agents against microbial pathogens, but are eco-friendly and safe (Rashad et al. 2022).

Plant probiotics are microorganisms able to improve food quality, promote plant growth, and increase crop yields. The term “Plant Probiotics (PPs)” can be used to decipher a distinct group of microbial strains with all the characteristics necessary to classify them as biofertilizers that affect plant growth through direct and indirect mechanisms (microbes that exhibit traits beneficial to plants in terms of growth and yield) (Rai et al. 2023). Agriculture primarily focuses on the mass production of grains and other food crops in a sustainable manner to produce food for an ever-growing market. Agricultural crop yields and other environmental factors lead to soil degradation, environmental pollution, and biodiversity. Plant growth-promoting microorganisms, also identified as "Plant Probiotics (PPs)", have gained recognition. As bio-elicitors, PPs promote plant growth and colonize soil or plant tissues when administered in soil, seeds, or plant surface and are used as an alternative means to avoid heavy use of agrochemicals (Upadhayay et al. 2023). In addition, agrochemicals have shown a significant increase in crop yields in the past few decades, but adverse effects have emerged later from overuse of chemical fertilizers. It led to the deterioration of soil quality, disturbance of the soil microbial environment, pollution of soil and water bodies, and adverse effects on human health due to pesticide and herbicide residues (Boregowda et al. 2022). The transition to organic farming, particularly the use of bio-fertilizers, has provided an environmentally friendly alternative to chemical-based farming, as well as improving crop yields and soil quality (Elnahal et al. 2022). Both the rhizosphere and the inner regions of plant tissues serve as a special hub for their respective microbial communities, the rhizomicrobiome, and endophytomicrobiome are a rich source of plant probiotics due to the many traits they possess, such as the production of the plant hormones IAA (Nazli et al. 2020), and lytic enzymes (Reddy et al. 2022). Plant probiotics enhance nutrient absorption and provide protection to plants from environmental stresses, such as biotic and abiotic stresses, as well as improve plant health (Pandey et al. 2022). Plant probiotics with diverse capabilities to stimulate plant growth provide benefits such as improved crop yield and food security (Ghoghari et al. 2022).

In *vivo*, the *A. marplatensis* is non-pathogenic (devoid of virulence factors) and capable of producing IAA and gibberellins and phosphorus solubilization ability. In *vitro*, inoculation of tomatoes with *A. marplatensis*, it resulted in a significant increase in vegetative growth, yield parameters and endogenous plant hormone content compared to the common free diazotrophic PGPR, *Azotobacter chroococcum* (Abdel-Rahman et al. 2017). Current projects on whole-genome sequencing of *B. velezensis* have identified a large number of biosynthetic gene clusters encoding enzymes for the synthesis of several antimicrobial compounds, however, its biological applications have not yet been identified or established. This bacterium is known to exert antagonistic effects against plant pathogens via production of diverse antimicrobial compounds (Fazle and Baek 2020).

Potato is a perennial plant of the *Solanaceae* family and one of the most commonly cultivated tuber crops, ranking fourth among the most important food crops in the world after major cereal crops, such as rice, wheat and maize. It can be one of the most important crops to be introduced in a region where the population suffers from frequent malnutrition due to heavy dependence on cereal crops and poor crop yields due to its ability to provide high yields of high quality product per unit input with shorter crop cycle compared to major cereal crops (Muleta and Aga 2019).

Root rot is one of the most important soil and seed borne fungal diseases. Endophytic bacteria and their natural bioproducts have emerged as growth promoters and disease inhibitors in the current era. Despite limited research, seeds are good purveyors of internal microbes, which are transmitted from them to other parts of the seedling, thus providing a shield against biotic and abiotic disturbance and promoting growth in early germination and later stages. Inner seed bacteria and their lytic enzymes, growth stimulants and antifungal molecules can stimulate growth and prevent the development of root rot disease at the same time. The isolation experiment from fenugreek seeds showed the presence of *Achromobacter* sp., which produce IAA, contain antifungal compounds (e.g., 2-butanol, 3, 3’-oxybis-), and reduce the growth of *Rhizoctonia solani* by 43.75%. Bacterial cells and/or supernatants improved the growth, physiology and yield performance of fenugreek plants, and effectively suppressed the progression of root rot disease under natural greenhouse and field conditions (Rashad et al. 2022).

One of the main diseases of potatoes during storage is dry rot, which is caused by many types of *Fusarium*. Biological control based on natural bioactive compounds that inhibit tuber disease offers an attractive alternative because it is environmentally friendly and low in toxicity. *Fusarium spp*., a pathogen of dry rot, significantly affects not only tuber yield and quality, but also produces mycotoxins. Common toxic species include *F. sambucinum, F. solani, F. oxysporum, F. culmorum, F. avenaceum, F. coeruleum* (Liu et al. 2022).

Potatoes are affected by one of the most common soil-borne pathogens, *R. solanacearum*, which causes wilt symptoms in plants and brown rot in potato tubers worldwide. Pathogens enter plants through various natural openings and wounds, and are easily spread by infected biological material, contaminated irrigation water, surface water, and soil. Pathogens can live for several years in association with alternative hosts. Indoor plants play a vital role in promoting plant growth and health. The healthy flora was further enriched with beneficial bacteria and bacteria with antagonistic activity against *R. solanacearum.* In healthy plants, abundant bacteria and biomarkers (*Pseudomonas* and *Streptomyces*) and essential microorganisms (*Bacillus, Lysobacter* and *Paenibacillus*) were plant-beneficial bacteria and showed antibacterial and plant growth-promoting activities. An endophytic strain *B. velezensis* produced bacillin to inhibit *R. solanacearum* (Li et al. 2023).

A broad range of synthetic agents produced by endophytes is widely used to balance the economic losses caused by pathogens in agriculture. Owing to their effect on nature and health risks, their application on a broader scale has been phasing out (Kim et al. 2017). The aim of this work to study the potentials of *A. marplatensis* and *B. velezensis* as plant probiotics for the field cultivation of potatoes.

## Materials and methods

### Probiotic bacterial

The most potent probiotic isolates used in the present work were identified using 16S rRNA gene sequence to provide genus and species for isolates by Sigma Scientific Services Company, Giza, Egypt. DNA extraction was carried out by using protocol of Gene Jet genomic DNA purification Kit, then PCR cleans up to the PCR product was done using Gene JET™ PCR Purification Kit. Sequences of 16S rRNA were matched with sequences of reference strains in a public repository NCBI GenBank database (http://www.ncbi.nlm.nih.gov) with accession numbers OQ457665 and OQ457694 were identified as *Achromobacter marplatensis* isolated from the *Matthiola incana* plant and *Bacillus velezensis* isolated from *Solanum tuberosum* plant, which were grown in sandy clay loamy soil and collected from the garden of the Atomic Energy Authority (AEA), Nasr City (East Cairo Suburb), National Center for Research and Radiation Technology (NCRRT), Al-Zomor Street, Nasr City, Cairo Government, Egypt. Probiotic isolates were stored by culturing them in a nutrient broth medium and placing them in a refrigerator at 4̊ C. Isolates were selected as potent plant probiotics because of their ability to produce IAA, a cellulase enzyme, and their antagonistic activities against the most common potato pathogens *R. solanacearum* and *F. oxysporum*.

### Performance of the selected plant probiotics at different conditions

The probiotic potential of *A. marplatensis* and *B. velezensis* was examined at a range of temperatures 25, 30, 35, and 40°C. The tested pH values were 5.5, 6.5, and 7.5. The tested probiotic potentials included IAA production, cellulase enzymatic activity, and antagonistic activity against *R. solanacearum* and *F. oxysporum*.

### Detection of IAA production under different conditions

For quantitative detection of IAA, endophytic bacterial isolates were grown in 20 ml of nutrient broth (NB) medium and incubated at 28°C for 48 h with vigorous shaking (100 rpm). The non-inoculated broth culture was used as the control. After incubation, IAA production was determined using the Salkowski method, according to Gordon and Weber (1951). The cultures were centrifuged at 2100 × g for 12 min, and 1 ml of the supernatant was transferred to a tube containing 2 ml of Salkowski reagent. The mixture was incubated in the dark for 30 min. The development of a red color was considered an indication of IAA production. The absorbance of the mixture was measured at 530 nm using a spectrophotometer. The concentration of IAA produced by the isolates was calculated using a standard curve constructed by measuring the absorbance at 0, 10, 20, 30, 40, 50, and 60 (μg/ml) Gordon and Weber (1951). The experiment was repeated three times.

### Detection of cellulase production under different conditions

The ability of the endophytic bacterial isolates to produce cellulase was tested. Screening for the cellulolytic activity of all isolates was carried out using Cellulose Congo Red agar media (CCA). (Hendricks et al. 1995) (g/l): K_2_HPO_4_ 0.50, MgSO_4_ 0.25, CMC 1.88, Gelatin 2.00, Congo red 0.20, Trace salt solution 1 ml (FeSO_4_.7H_2_O 0.1 ml–MnCL_2_.4H_2_O 0.1 ml–ZnSO_4_.7H_2_O 0.1 ml–distilled water 100) agar 20. The isolates were inoculated in plates using a sterile corkborer with a zone scale of 5 mm in diameter.

The plates were incubated at 37 °C for five days, and then the surface of each plate was flooded with 1M NaCl (58.5 g/L). Clear hydrolysis zones indicate carboxymethyl cellulose degradation. Cellulase activity was determined by measuring the ratio of the clear zone diameter to the colony diameter (Wang et al. 2003). The experiment was repeated three times.

### Antifungal activity of probiotic isolates under different conditions against *R. solanacearum* and *F.oxysporum*

The antagonistic activities of endophytic bacteria grown on nutrient agar media (NA media) (Lapage et al. 1970) (g/l): beef extract 1.0, yeast extract 2.0, peptone 5.0, NaCl 5.0, agar 15.0, pH 7.0, incubated at 30°C for 24 h were separately tested against *R. solanacearum* and *F. oxysporum*.

Nutrient agar plates inoculated with 1 ml bacterial suspension of *R. solanacearum* were used to screen for antibacterial activity (Mayr-Harting et al. 1972).

Saboraud dextrose agar (SDA) (Sabouraud 1892) (g/l): casein 5.0, dextrose 40.0, agar 15.0, and pH 5.6 at 28°C for 7 days, plates containing 1 ml spore suspension of *F. oxysporum* were used to screen for antifungal activity (Ullah et al. 2018).

All inoculations were prepared using the pour-plate method. A rounded disk 5 mm in diameter was removed from the center of each plate using a sterile cork borer. Each well was then filled with 50 µL of endophytic bacterial suspension. For the detection of antifungal activity, the plates were incubated at 28 °C for 4 days, whereas for the detection of antibacterial activity, the plates were incubated at 30 °C for 2 days. Control plates were prepared by adding 50 µL of 0.9% sterile saline solution to the wells instead of the endophyte suspensions. After incubation, clear zones around the inoculated wells were detected and measured. The experiment was repeated three times.

### Effect of ultraviolet (UV) radiation on selected isolates

The endophytic bacterial suspension in the saline solution was (Ultraviolet) irradiated in open Petri dishes in the dark. The Petri plates were placed under a 365 W.L UV lamp (Radiation) at a distance of 30 cm (emitting energy of 1. × 102 J/m^2^/s) for 1, 2, and 3 h. The dose response to radiation was determined directly by plating 1 ml of the bacterial suspension exposed for 1, 2, and 3 h separately onto nutrient agar plates to count the number of colony-forming units (CFU) after radiation exposure at each dose. Unirradiated plates served as controls. After 24 h, the plates were examined for bacterial counts, and the number of CFU at each dose was plotted against the corresponding dose. The experiment was repeated three times.

### In vivo application of plant probiotics for the cultivation of *Solanum tuberosum*

Pot culture experiments were conducted in a greenhouse at the experimental farm of NCRRT, Atomic Energy Authority, Cairo government, Egypt. The application of selected plant probiotics to follow up the effect of the most potent endophytic bacterial isolates for growth promotion, biological control, and biodegradation abilities (arranged with three replicates) on the potato plant (*S. tuberosum*) cultivated in sandy clay loamy soil, was collected for the in *vivo* study from the surface layer (0–40 cm) of NCRRT, Cairo, Egypt. Physical analysis of sandy clay loamy soil was carried out using the pipette method (FAO Soil Bulletin 1982), and chemical analysis (Jackson 1958) of the sandy clay loamy soil used (Jennison et al. 1985). Physical Properties of sand clay loamy soil: 25% clay, 21.5% silt and 53.5%. The chemical characteristics of sand clay loamy soil: Na^+^ 10.5, K^+^ 0.6, Ca^+2^ 12.3, CO_3_^−2^ 1.2, HCO^−3^ 4.40, CL^−^ 6.50, Mg^+2^ 3.6 and SO_4_^−2^ 13.5 with pH 7.62 and EC (ds m^−1^) 1.8

### Sources of isolated endophytic bacteria

Two endophytic isolates, *A. marplatensis* and *B. velezensis*, were selected as promising plant probiotics owing to their high growth-promoting hormone production, biological control, and biodegradation abilities.

### Growth conditions

Plastic bag pots (40.0 cm height, 20.0 cm diameter) were arranged under greenhouse conditions in *January* 2022 using *S. tuberosum* as a non-leguminous plant. Cultivated sandy clay loamy soil was packed in 72 pots, each one containing 9.0 kg soil and five randomly selected seeds from potatoes (approximately 50g weight with two growing buds for each seed) at the time of harvest. All agricultural practices were considered. Each pot was irrigated with 756 ml/pot of tap water daily according to the water-holding capacity (WHC) when needed.

### Inoculants

The most potent bacterial isolates, *A. marplatensis* and *B. velezensis* were grown separately in a nutrient broth and incubated at 28°C for 48 h. Twenty milliliters of broth media containing approximately 10^3^ cfu/ml from this bacterial isolate, (by using the serial dilution method and confirmed by measuring turbidity and adjusting it with a spectrophotometer) which grew on nutrient broth media, was added to each pot in the first group from the experiment once, and 5 g of wheat was added, which was inoculated with the bacterial isolates, and incubated at 28 °C for 48 h to each pot in the second group from experiment one time. In addition, 20 ml of *F. oxysporum* spores containing approximately 103 cfu/ml were added as pathogenic fungi that grew on SAD media incubated at 28 °C for 7 days to each pot in the first group from the experiment once, and 5 g of wheat inoculated with the pathogenic fungus *F*. *oxysporum*, which was incubated at 28 °C for 7 days in the second group. In addition, 20 ml of *R. solanacearum* spores containing approximately 103 cfu/ml was added as pathogenic bacteria that grew on nutrient agar media and incubated at 30 °C for 24 h in each pot in the first group from the experiment once, and 5 g of wheat inoculated with the pathogenic bacteria *R. solanacearum*, which was incubated at 30 °C for 24 h to each pot in the second group from experiment also one time.

The treatments were arranged in three replicates and split into two groups:

Two groups of pots were treated with one type of inocula. The following treatments were applied to each group (triplicate pots were used for each treatment):

- Uninoculated (control) potato seeds without probiotics.
- Potato seeds inoculated with *A. marplatensis*.
- Potato seeds inoculated with *B. velezensis*.
- Potato seeds inoculated with [*A. marplatensis* + *B. velezensis*].
- Uninoculated (control) infected with *R. solanacearum* as pathogenic bacteria.
- Potato seeds inoculated with *A. marplatensis* infected with *R. solanacearum*.
- Potato seeds inoculated with *B. velezensis* infected with *R. solanacearum*.
- Potato seeds inoculated with [*A. marplatensis* + *B. velezensis*] infected with *R. solanacearum*.
- Uninoculated (control) infected with *F. oxysporum* as pathogenic Fungi.
- Potato seeds inoculated with *A. marplatensis* infected with *F. oxysporum*.
- Potato seeds inoculated with *B. velezensis* infected with *F. oxysporum*.
- Potato seeds inoculated with [*A. marplatensis* + *B. velezensis*] infected with *F. oxysporum*.

At maturity, after 90 d, *S. tuberosum* plants were harvested. A number of morphological and yield parameters were recorded, including wet and dry weight in grams, length of shoots and roots in centimeters, and the number of branches and leaves counted. Shoot and root rot was detected, the number of tubers was counted, and the weight was determined in grams.

### Statistical analysis

Experiments were carried out in triplicates. All experimental data were subjected to ANOVA analysis to estimate the least significant differences (LSD) at *P* ≤ 0.05 to compere the variation between treatments. Duncan’s multiple range test (DMRT) was followed for comparison between significant means at probability 0.05 according to Walter and Duncan (1969) using MSTAT-C program software version 1.42. The data which have different letters, within each assay, were significantly different.

## Results

### Performance

The purpose was to determine the optimal conditions for the production of IAA and cellulase, and the biological resistance of pathogenic organisms, such as *F. Oxysporum* and *R. solanacearum*, by determining the optimal temperature and pH for the growth of the selected endophytic bacterial isolates, which were defined as A. *marplatensis* and *B. velezensis*.

### Performance of IAA production

**Fig. 1:**
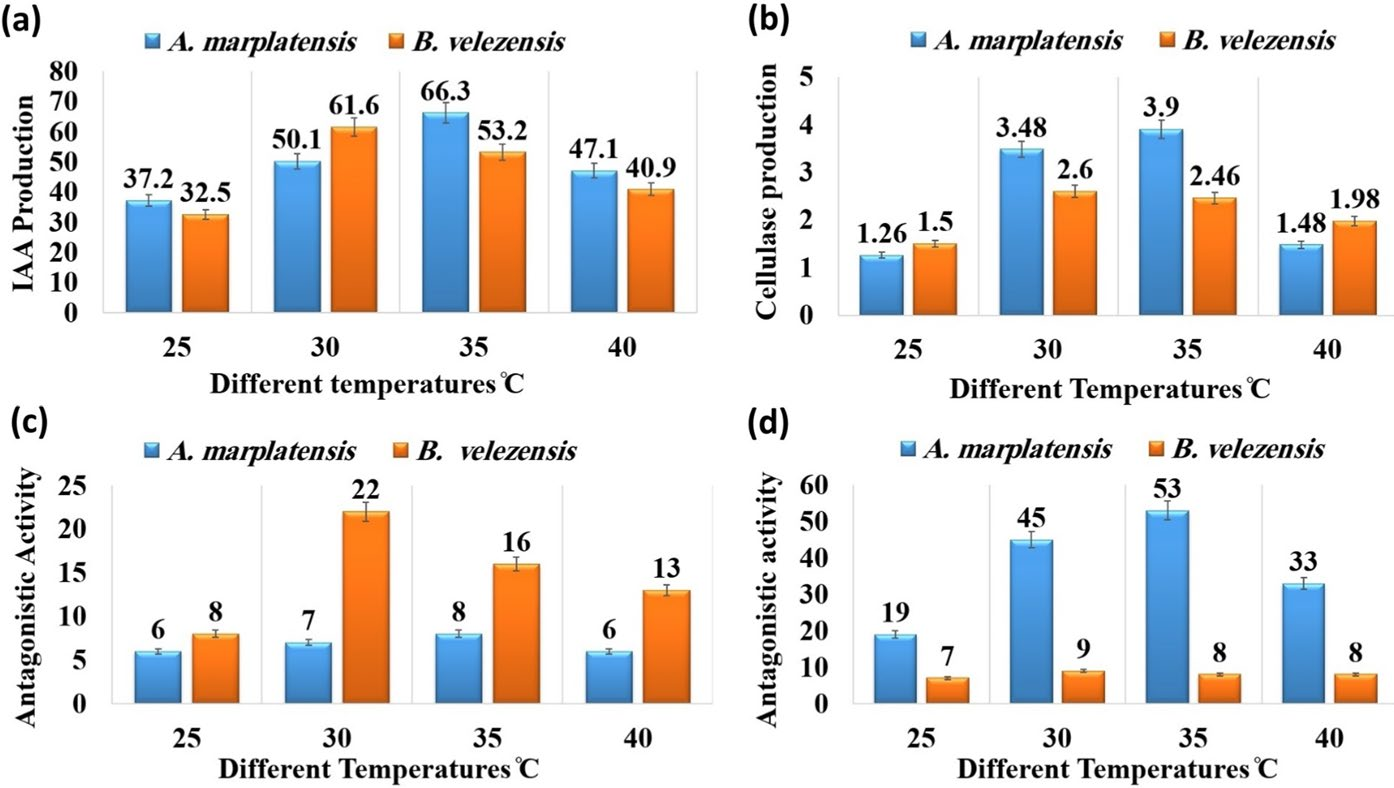
Showed performance to determine the optimal temperature. **a** Performance of IAA production by A. *marplatensis* and *B. velezensis* at different temperatures. **b** Performance of cellulase production by A. *marplatensis* and *B. velezensis* at different temperatures. **c** Antagonistic activity effect against *R. solanacearum* by A. *marplatensis* and *B. velezensis* at different values from pH. **d** Antagonistic activity effect against *F. oxysporum* by A. *marplatensis* and *B. velezensis* at different temperatures

**Fig. 2:**
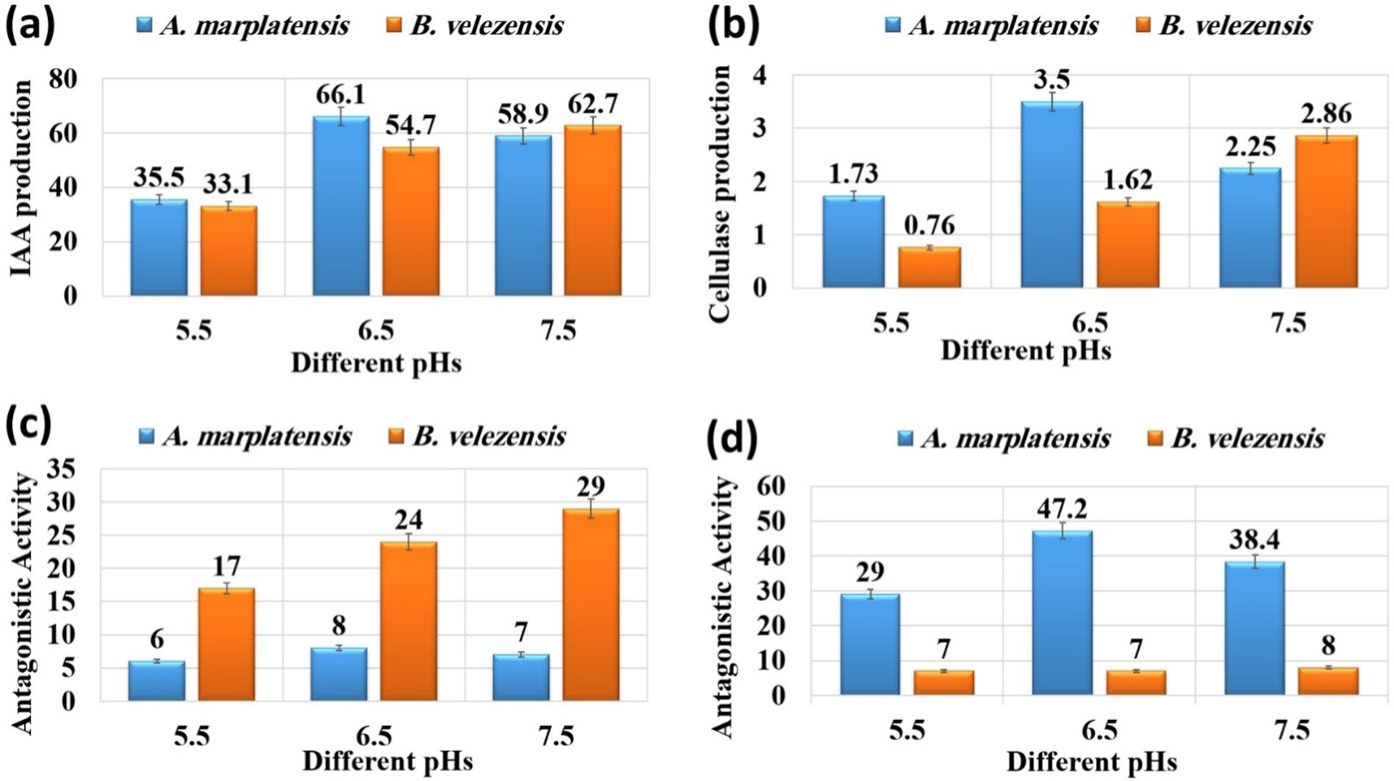
Showed performance to determine the optimal pH. **a** Performance of IAA production by A. *marplatensis* and *B. velezensis* at different values from pH. **b** Performance of cellulase production by A. *marplatensis* and *B. velezensis* at different values from pH. **c** Antagonistic activity effect against *R. solanacearum* by A. *marplatensis* and *B. velezensis* at different values from pH. (d) Antagonistic activity effect against *F. oxysporum* by A. *marplatensis* and *B. velezensis* at different values from pH

The optimum parameters for IAA production from the efficient isolates *A. marplatensis* and *B. velezensis* were determined and recorded. After incubation at different temperatures of 25, 30, 35, and 40 °C, it was noticed that the optimum temperature for *A. marplatensis* was observed at 35 °C whereby significantly the maximum yield of 66.3, and 61.6 ug/ml was observed at 30 °C by *B. velezensis* as shown in (Fig. 1a). The optimum pH for *A. marplatensis* was observed at pH 6.5, whereby the maximum yield was 66.1, and 62.7 ug/ml was observed at pH 7.5, as shown in (Fig. 2a) On the other hand, significantly it was found that production of IAA by *A. marplatensis* was better than *B. velezensis*.

### Performance of cellulase production

The optimum parameters for cellulase production from the probiotics *A. marplatensis* and *B. velezensis* were determined and recorded. The optimum temperature for cellulase activity was 35 °C for *A. marplatensis* 3.90 mm and *B. velezensis* 2.6 mm at 30°C, which showed the highest enzyme activity (Fig. 1b). It was noticed that the optimum pH of 6.5 for *A. marplatensis* and *B. velezensis* significantly was observed at pH 7.5 whereby maximum yield 3.50 and 2.86 mm respectively as shown in (Fig. 2b). On the other hand, significantly it was found that the production of cellulase enzyme by *A. marplatensis* was better than *B. velezensis*.

### Effect of antagonistic activity *R. solanacearum*

The results indicated that the optimum temperature for the antagonistic activity effect against pathogen *R. solanacearum* was at 35 °C for *A. marplatensis* 8 mm and *B. velezensis* 22 mm at 30 °C, which showed the highest antagonistic activity effect, as shown in (Fig. 1c). The results indicated that the optimum pH for antagonistic activity against *R. solanacearum* was pH 6.5 for *A. marplatensis* 8 mm and *B. velezensis* 29 mm, which showed the highest antagonistic activity effect, as shown in (Fig. 2c). On the other hand, significantly it was found that the antagonistic activity by *B. velezensis* was better than *A. marplatensis*.

### Antagonistic activity effect against *F. oxysporum*

The optimum temperature for antagonistic activity against *F. oxysporum* was 35 °C for *A. marplatensis* 53 mm and *B. velezensis* 9 mm at 30 °C, and *A. marplatensis* showed the highest antagonistic activity after incubation for 24 h (Fig. 1d). The optimum pH for the antagonistic activity against *F. oxysporum* significantly was pH 6.5 for *A. marplatensis* 47.2 mm and *B. velezensis* 8 mm, where *A. marplatensis* showed the highest antagonistic activity effect, as shown in (Fig. 2d). On the other hand, significantly it was found that the antagonistic activity by *A. marplatensis* was better than *B. velezensis*.

### Effect of radiation doses ultraviolet (UV)

**Fig. 3:**
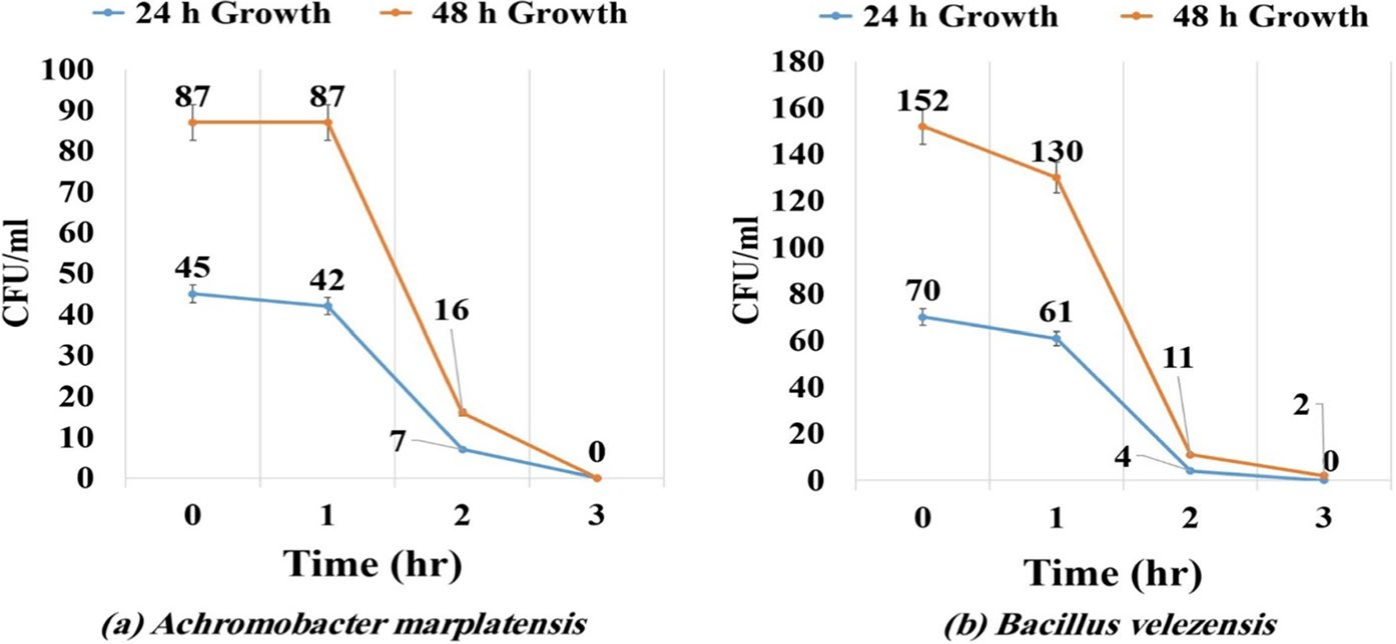
Survival of isolates upon UV exposure for different periods **a** *Achromobacter marplatensis* **b** *Bacillus velezensis*

The aim of the experiment is to determine the ability of bacteria to survive in the field and resist radiation during the day in vivo. According to *A. marplatensis* and *B. velezensis* which incubated for 24 and 48 h, the results illustrated in (Fig. 3a, b) indicate that significantly a gradual decrease in bacterial count was observed after exposure to UV radiation at 1 and 2 h while no noticeable growth rate was observed when exposed for 3 h compared to the control (without exposure to radiation).

### Greenhouse experiment in vivo

#### Effect of adding promising plant probiotics on the growth of *S. tuberosum* plants

##### Probiotics + Potato seeds infected with *R. solanacearum* as the pathogen

**Fig. 4:**
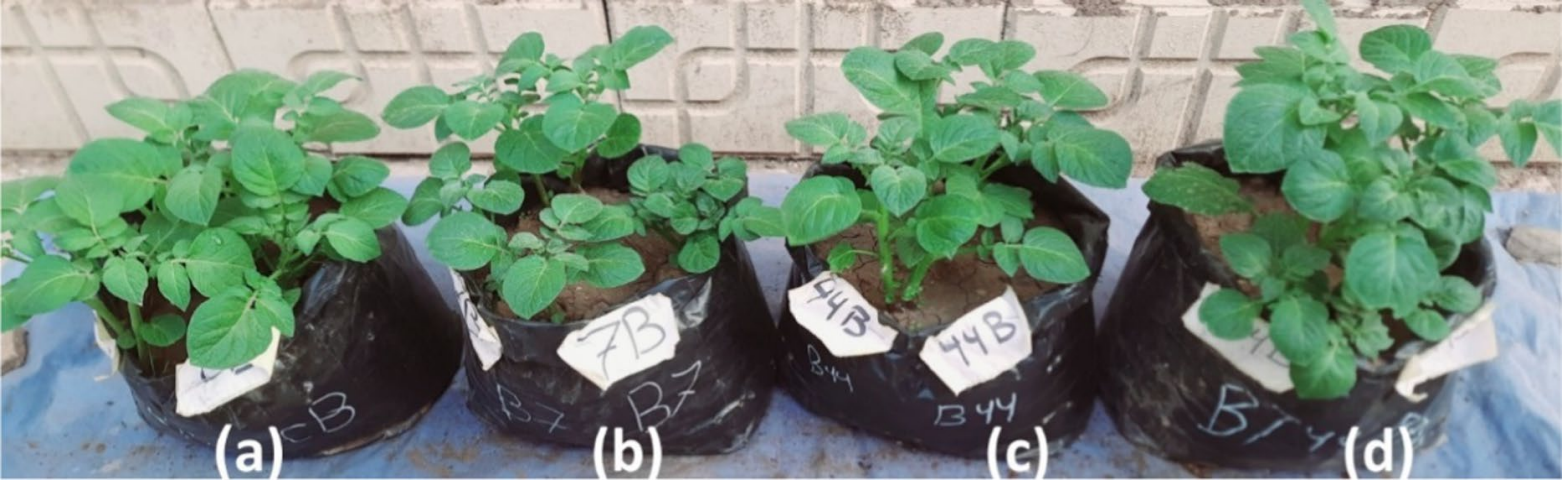
Potato plants infected with *R. solanacearum* as the pathogen loaded on broth media. **a** Potato plants inoculated with pathogenic bacteria *R. solanacearum* loaded on broth media (Control). **b** Potato plants inoculation with [*A. marplatensis* + *R. solanacearum*] loaded on broth media. **c** Potato plants inoculated with [*B. velezensis* + *R. solanacearum*] loaded on broth media. **d** Potato plants inoculated with [*A. marplatensis* + *B. velezensis* + *R. solanacearum*] loaded on broth media

**Fig. 5:**
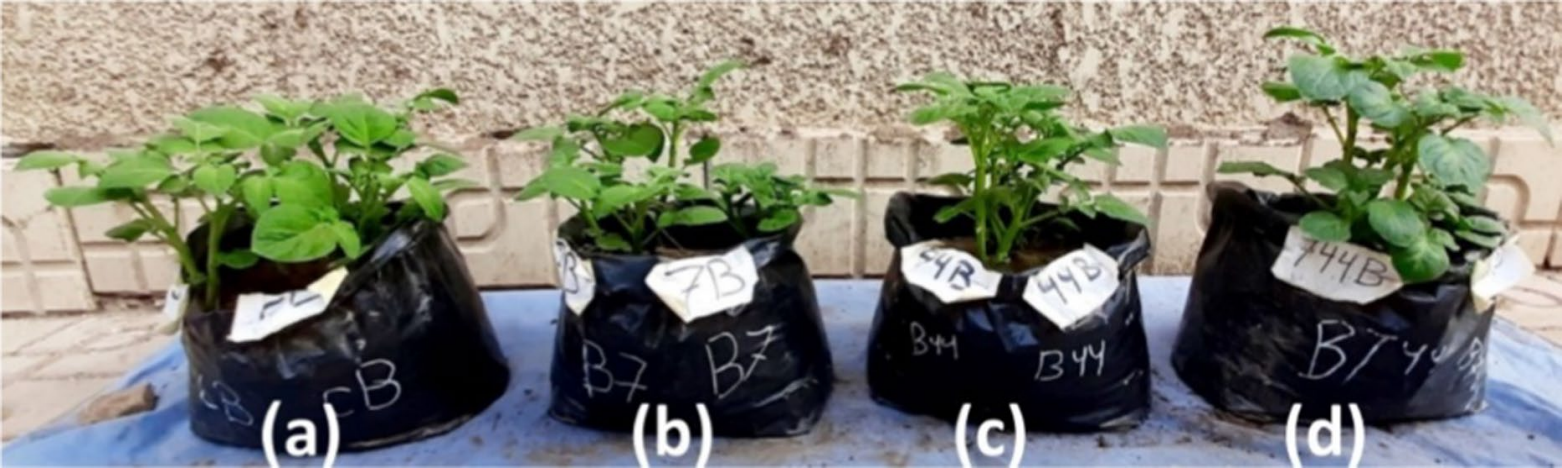
Potato plants infected with *R. solanacearum* as the pathogen loaded on wheat grains. **a** Potato plants inoculated with pathogenic bacteria *R. solanacearum* loaded on wheat grains (Control). **b** Potato plants inoculation with [*A. marplatensis* + *R. solanacearum*] loaded on wheat grains. **c** Potato plants inoculated with [*B. velezensis* + *R. solanacearum*] loaded on wheat grains. **d** Plants inoculation with [*A. marplatensis* + *B. velezensis* + *R. solanacearum*] loaded on wheat grains

The antagonistic effect of *A. marplatensis* and *B. velezensis* on *S. tuberosum* shoots artificially infected with *R. solanacearum* as a pathogen was detected after cultivation for 90 days. The results shown in (Table 1) exhibit remarkable protective effects of *A. marplatensis* and *B. velezensis* against *R. solanacearum*, either if added to the cultivation soil on broth media (Fig. 4) or in wheat grains (Fig. 5). Soil artificially infected with *R. solanacearum* resulted in a reduction in wet weight, dry weight, shoot length, number of branches, leaves, root length, total tuber weight, and the number of tubers. The shoot rot percentage was reduced from 100 to 25% when both probiotics were added in wheat grains to the cultivation soil either separately or together, whereas the rot percentage of the shoots was 0% when the probiotics were added to the soil carried on the broth protective significantly. The best effect of using both probiotics together against *R. solanacearum* in broth exceeded that of using them separately or with wheat grains, indicating the synergistic effect of both probiotics against *R. solanacearum.* Forms of potatoes infected with *R. solanacearum* was presented in (Fig. 8a).

**Table 1:** Effect of probiotics (*A. marplatensis* and *B. velezensis*) on the growth of potato plants artificially infected with *R. solancerium* in sandy clay loamy soil for 90 days

Different Bio agents	Disease parameters		Growth characters								
	Survival plants	Disease index	Shoot				Root				
	Leaves No	Root Rot%	Wet wt. (gm)	Dry wt. (gm)	Length (cm)	Branches	Wet wt. (gm)	Dry wt. (gm)	Length (cm)	Tuber wt	Tuber No
Control (1): *R. solancerium* (broth)	79 ± 9^i^	100%	34.6 ± 2^de^	11.5 ± 1^c^	13 ± 3^h^	26 ± 1^j^	22 ± 2^a^	6.8 ± 3^de^	22.8 ± 3^hi^	9.0 ± 2^c^	7 ± 1^de^
Control (2): *R. solancerium* (wheat grains)	75 ± 5^g^	100%	32.6^c^	9.8 ± 1^a^	8.9 ± 0^ab^	24 ± 2^ef^	20 ± 1^a^	6.3 ± 2^cde^	21.3 ± 1^cd^	3.5 ± 1^bc^	4 ± 1^cde^
*A.marplatensis* (broth) infected with *R. solancerium*	104 ± 4^fg^	25%	62.7 ± 2^de^	21.1 ± 1^cd^	18.3 ± 2^c^	34 ± 4^cd^	27.8 ± 2^b^	9.5 ± 2^ab^	23.8 ± 2^ef^	13.0 ± 2^bc^	11 ± 1^ab^
*A.marplatensis* (wheat grains) infected with *R. solancerium*	95 ± 5^c^	25%	54.5 ± 4^bc^	17.3 ± 2^bc^	17.3 ± 3^bc^	29 ± 4^c^	26.2 ± 3^b^	8.3 ± 1^a^	23.3 ± 3^a^	15.5 ± 2^bc^	9 ± 2^a^
*B.velezensis* (broth) infected with *R. solancerium*	99 ± 3^b^	0%	59.0 ± 3^a^	19.6 ± 1.3^b^	17.3 ± 1^b^	29 ± 2^b^	26.5 ± 1^c^	8.3 ± 1^bcd^	23 .0 ± 1^b^	9.0 ± 3^c^	8 ± 2^bcd^
*B.velezensis* (wheat grains) infected with *R. solancerium*	93 ± 3^ef^	50%	48.8 ± 2^cd^	15.6 ± 1^bc^	15.6 ± 2^de^	26 ± 3^a^	25.8 ± 3^d^	7.5 ± 1^bcd^	23.0 ± 1^d^	7.8 ± 1^b^	7 ± 2^bcd^
[*A. marplatensis* + *B. velezensis*] (broth) infected with *R. solancerium*	137 ± 2^i^	0%	84.3 ± 4^hi^	26.8 ± 2^ghi^	21.5 ± 1^de^	36 ± 6^efg^	34.7 ± 4^d^	11.9 ± 2^e^	27.9 ± 1^cd^	36.4 ± 3^ab^	18 ± 1^e^
[*A. marplatensis* + *B. velezensis*] (wheat grains) infected with *R. solancerium*	125 ± 1^hi^	25%	82.3 ± 2^efg^	24.8 ± 2^fgh^	19.3 ± 2^cd^	36 ± 2^i^	29.9 ± 2^d^	9.9 ± 1^a^	25.3 ± 2^c^	33.7 ± 1^cd^	15 ± 3^b^
Mean	100.9	40.6	57.4	18.3	16.4	30	26.7	8.6	23.8	16	10
LSD (0.05)	Leaves no.T:2.045, F: 1.174, TF 2.535—Shoot wet wt. T: 1.053, F: 1.653, TF: 2.095—Shoot dry wt. T: 0.157, F: 0.122, TF: 0.263—Shoot length: T: 0.183, F: 0.123, TF: 0.266—Branches: T: 0.182, F: 0.283. TF: 0.479—Root wet wt. T: 0.067, F: 0.58, TF: 0.164—Root dry wt. T: 0.352, F: 0.243, TF: 0.762—Root length: T: 0.395, F: 0.713, TF: 1.156—Tuber wt. T: 1.047, F: 1.478, TF: 2.074—Tuber No. T: 0.014, F: 0.025, TF: 0.078 The data are the mean of three replicates ± SE Means having the same letter are not significantly different using Duncan’s multiple range test (DMRT) (*P* < 0.05)

##### Probiotics + Potato seeds infected with *F. oxysporum* as the pathogen

**Fig. 6:**
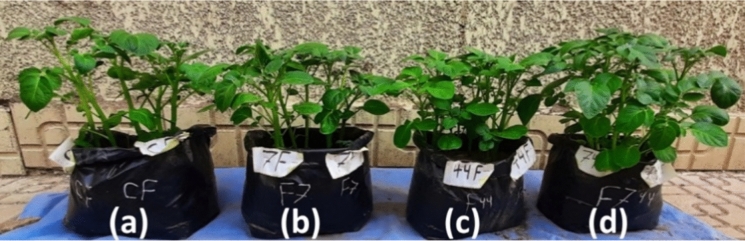
Potato seeds infected with *F. oxysporum* as the pathogen loaded on broth media. **a** Potato plants inoculated with pathogenic Fungi (*F*. *oxysporum*) loaded on broth media (Control). **b** Potato plants inoculated with [*A*. *marplatensis* + *F*. *oxysporum*] loaded on broth media. **c** Potato plants inoculated with [*B. velezensis* + *F*. *oxysporum*] loaded on broth media. **d** Potato plants inoculated with [*A*. *marplatensis* + *B*. *velezensis* + *F*. *oxysporum*] loaded on broth media

**Fig. 7:**
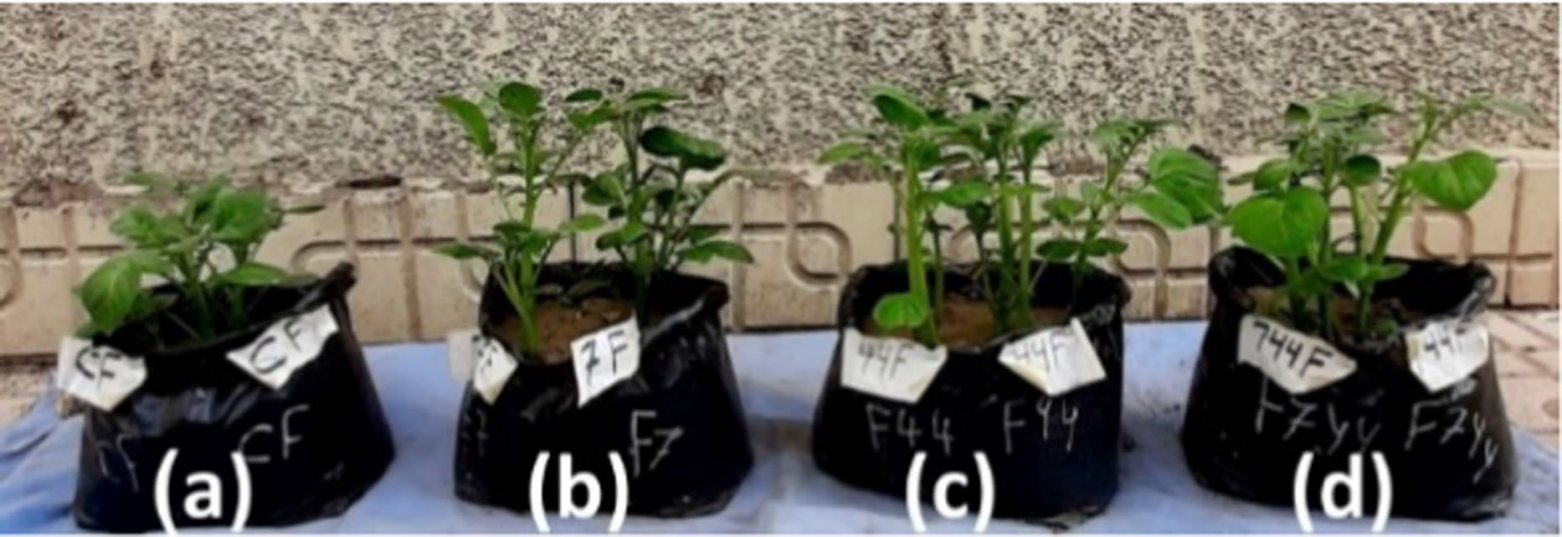
Potato seeds infected with *F. oxysporum* as the pathogen loaded on wheat grains. **a** Potato plants inoculated with pathogenic Fungi (*F. oxysporum*) loaded on wheat grains (Control). **b** Potato plants inoculated with [*A*. *marplatensis* + *F. oxysporum*] loaded on wheat grains. **c** Potato plants inoculated with [*B*. *velezensis* + *F. oxysporum*] loaded on wheat grains. **d** Potato plants inoculated with [*A*. *marplatensis* + *B*. *velezensis* + *F. oxysporum*] loaded on wheat grains

**Fig. 8:**
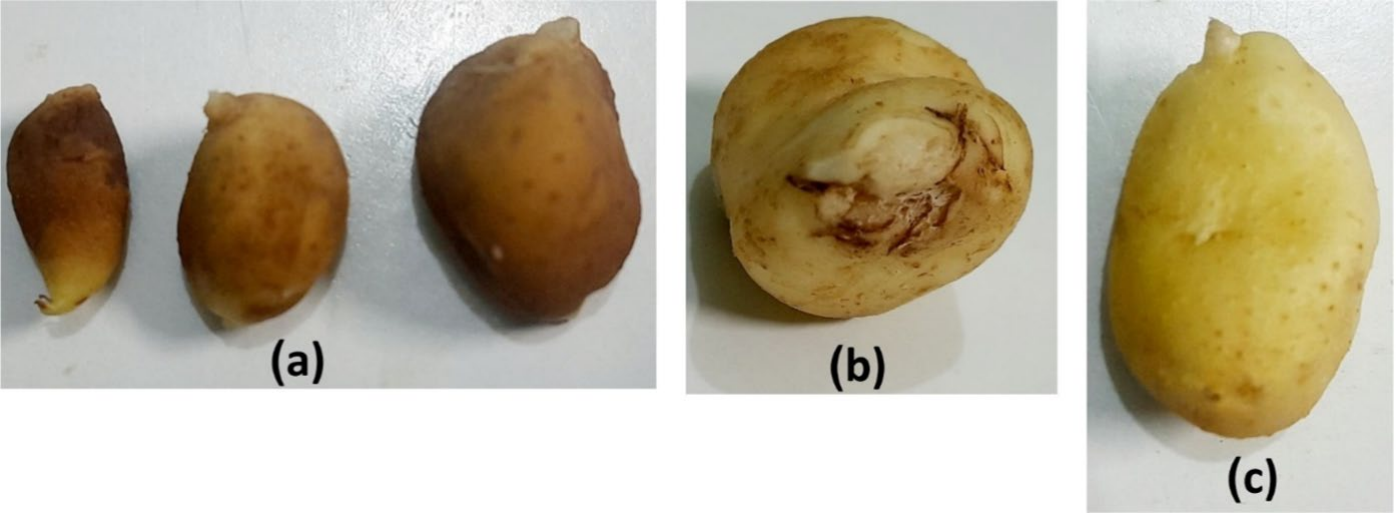
Different forms of infected potatoes. **a** Potatoes infected with *R. solanacearum* (bacterial wilt diseases). **b** Potatoes infected with *F. oxysporum* (*Fusarium* wilt diseases). **c** Production of potatoes without the appearance of diseases using probiotics *A*. *marplatensis* and *B*. *velezensis*

The results in (Table 2) show that the shoot rot percentage was reduced from 100 to 0% when both probiotics were added in broth (Fig. 6) or wheat grains (Fig. 7) to the cultivation soil either separately or together. The effect of using both probiotics together against *F. oxysporum* exceeded using them separately, indicating the synergistic effect of both probiotics against *F. oxysporum.* The root rotting percent in the infected pots was 75% compared to 0% in the control. The root rot percentage was reduced from 100 to 0% when both probiotics were added in broth to the cultivation soil either separately or together while the rot percent of the roots was less than 30% when the probiotics were added to the soil carried on wheat grains protective significantly. Forms of potatoes infected with *F. oxysporum* was presented in (Fig. 8b). The production of potatoes without the appearance of diseases using probiotics *A*. *marplatensis* and *B*. *velezensis* which presented in (Fig. 8c) and (Table 3).

**Table 2:** Effect of probiotics (*A. marplatensis* and *B. velezensis*) on the growth of potato plants artificially infected with *F. oxysporum* in sandy clay loamy soil for 90 days

Different bio agents	Disease parameters		Growth characters								
	Survival plants	Disease index	Shoot				Root				
	Leaves No	Root Rot%	Wet wt. (gm)	Dry wt. (gm)	Length (cm)	Branches	Wet wt. (gm)	Dry wt. (gm)	Length (cm)	Tuber wt	Tuber No
Control (1): *F. oxysporum* loaded in broth	94 ± 4^b^	75%	89.5 ± 2^a^	26.7 ± 2^c^	25 ± 1^h^	38 ± 3^ef^	27 ± 1^de^	6.8 ± 1^de^	27 ± 1^a^	4.8 ± 1^fg^	7 ± 2^b^
Control (2): *F. oxysporum* loaded in wheat	91 ± 1^a^	75%	84 ± 4^a^	24.4 ± 2^a^	23.3 ± 2^ab^	38 ± 1^j^	27.3 ± 2^c^	6.8 ± 2^cde^	26.3 ± 2^fg^	2 ± 1^c^	4 ± 1^a^
*A.marplatensis* (broth) infected with *F. oxysporum*	127 ± 2^cde^	0%	158 ± 3^b^	48.6 ± 4^cd^	35.3 ± 2^c^	59 ± 2^cd^	37.7 ± 1^de^	10.5 ± 1^ab^	33.6 ± 3^i^	25 ± 3^b^	18 ± 2^bc^
*A.marplatensis* (wheat grains) infected with *F. oxysporum*	103 ± 3^ab^	25%	152 ± 1^b^	42.6 ± 2^bc^	33.3 ± 3^bc^	56 ± 2^c^	32.7 ± 2^bc^	8.8 ± 2^a^	32 ± 1^c^	22 ± 2^a^	14 ± 1^e^
*B. velezensis* (broth) infected with *F. oxysporum*	122 ± 2^a^	0%	155 ± 5^c^	43 ± 3^a^	34.4 ± 4^b^	58 ± 4^c^	35.3 ± 1^a^	9.7 ± 1^bcd^	32 ± 2^d^	23 ± 3^cd^	17 ± 2^g^
*B. velezensis* (wheat grains) infected with *F. oxysporum*	99 ± 1^bcd^	25%	137 ± 3^d^	36 ± 0.3^cd^	24.3 ± 2^de^	54 ± 4^b^	31.3 ± 1^cd^	7.2 ± 2^bcd^	27 ± 2^de^	20.9 ± 1^bc^	16 ± 2^c^
[*A. marplatensis* + *B. velezensis*] (broth) infected with *F. oxysporum*	130 ± 5^bc^	0%	175 ± 5^d^	50 ± 1.2^hi^	45.7 ± 2^de^	70 ± 5^efg^	44.2 ± 2^hi^	12.9 ± 2^e^	35.7 ± 2^bc^	37 ± 2^b^	24 ± 4^d^
[*A. marplatensis* + *B. velezensis*] (Wheat grains) infected with *F. oxysporum*	122 ± 2^e^	25%	163 ± 3^c^	49 ± 3^efg^	36.3 ± 2^cd^	62 ± 2^hi^	38.3 ± 2^efg^	10.7 ± 0.2^a^	32.3 ± 2^b^	24 ± 0.2^b^	18 ± 1^d^
Mean	111	28	139.1	40	32.2	54.4	34.2	9.17	30.7	19.8	14.7
LSD (0.05)	Leaves no.T:2.043, F: 1.133, TF 2.132—Shoot wet wt. T: 1.065, F: 1.553, TF: 2.045—Shoot dry wt. T: 0.185, F: 0.125, TF: 0.286—Shoot length: T: 0.148, F: 0.196, TF: 0.265—Branches: T: 0.186, F: 0.245. TF: 0.345—Root wet wt. T: 0.034, F: 0.245, TF: 0.267—Root dry wt. T: 0.462, F: 0.353, TF: 0.661—Root length: T: 0.465, F: 0.653, TF: 1.276—Tuber wt. T: 1.067, F: 1.345, TF: 2.105—Tuber No. T: 0.023, F: 0.029, TF: 0.067 The data are the mean of three replicates ± SE Means having the same letter are not significantly different using Duncan’s multiple range test (DMRT) (*P* < 0.05)

## Discussion

This study aimed to investigate the production of IAA hormone, cellulase enzyme, and biological resistance to potato pathogens such as *F. Oxysporum* and *R. solanacearum* by knowing the best temperature and pH for the growth of selected bacterial isolates *A. marplatensis* and *B. velezensis.* The optimum temperature for *A. marplatensis* and *B. velezensis* was observed at 35 and 30 ^°^C whereby the maximum yield of IAA 66.3 and 61.6 ug/ml respectively, while the optimum pH was observed at pH 6.5 and 7.5 whereby maximum yield 66.1 and 62.7 ug/ml respectively.

Jiménez-Gómez et al. (2017) reported that, the highest production of IAA was shown by *Achromobacter sp.* 13.50 μg/mg after incubation at 30°C and pH 7 for 4 days. Kumari et al. (2018) showed that, 76.41% of endophytic bacterial isolates produced indole acetic acid by *Achromobacter sp.* at 37°C and pH 7 for 5 days. Abo Elsoud et al. (2023) mentioned that, maximum yield of IAA 56.60 μg/mL at 28.5 °C and 7.69 pH. *B. velezensis* can be used to increase soil fertility or IAA production to improve plant growth.

The optimal conditions for cellulase enzyme production by the efficient isolates *A. marplatensis* was 3.90 mm observed at 35 °C while at 30 °C was 2.6 mm for *B. velezensis.* The optimum pH was 6.5 for *A. marplatensis* and 7.5 for *B. velezensis* were observed by the maximum yield 3.50 and 2.86 mm respectively. Mousa et al. (2019) reported that, the optimum pH value was 7 at 35 °C for *Achromobacter spanius*. Sarangthem et al. (2023) reported that, the optimum temperature and pH for cellulase activity were at 55 °C and pH 7.0 by *B. velezensis*. The enzyme is stable over a wide range of temperatures from 30 to 70 °C with maximum activity observed at 48 h of incubation.

**Table 3:** Effect of Probiotics (*A. marplatensis* and *B. velezensis*) on the growth of potato plants cultivated in sandy clay loamy soil for 90 days

Different bio agents	Disease parameters		Growth characters								
	Survival plants	Disease index	Shoot				Root				
	Leaves No	Root Rot%	Wet wt. (gm)	Dry wt. (gm)	Length (cm)	Branches	Wet wt. (gm)	Dry wt. (gm)	Length (cm)	Tuber wt	Tuber No
Control: plant without probiotics or pathogens	99 ± 6^de^	0%	84.7 ± 2.3^a^	28.5 ± 1.3^d^	24.3 ± 1.2^e^	40 ± 4^de^	29.2 ± 2^a^	9.3 ± 1.7^d^	24 ± 5.8^bcd^	15.1 ± 2.3^c^	9 ± 1^c^
*A.marplatensis* (broth)	179 ± 9^cde^	0%	112.3 ± 5.2^a^	37.4 ± 2.6^d^	27.4 ± 1.5^c^	55 ± 5^b^	34.5 ± 1.9^a^	11.0 ± 1^d^	26 ± 1^e^	26.5 ± 3.4^d^	16 ± 2^cd^
*A.marplatensis* wheat grains	149 ± 1^ab^	0%	89.6 ± 3.5^b^	30.3 ± 4^de^	24.3 ± 1^de^	47 ± 2^c^	31.5 ± 1^b^	10.3 ± 1^de^	24 ± 4^a^	15.1 ± 1^de^	22 ± 2^ab^
*B. velezensis* (broth)	157 ± 1^a^	0%	108.5 ± 1^b^	35.8 ± 2^cde^	25.8 ± 1^a^	42 ± 2^d^	33.3 ± 1^b^	11.3 ± 1^cde^	25.7 ± 2^a^	22.7 ± 1^cde^	12 ± 1^ab^
*B. velezensis* wheat grains	124 ± 4^bcd^	0%	86.7 ± 1^c^	29.5 ± 1^ab^	22.9 ± 2^cde^	40 ± 0^d^	29.7 ± 1^c^	10.1 ± 1^ab^	23 ± 3^c^	16.5 ± 1^cde^	9 ± 0^e^
[*A. marplatensis* + *B. velezensis*] (broth)	276 ± 6^bcd^	0%	140.3 ± 0.3^d^	47.7 ± 2^a^	32.3 ± 1.5^e^	65 ± 0^de^	43.7 ± 8.7^d^	16.4 ± 0.4^a^	28.6 ± 3^b^	40.8 ± 2^bcd^	20 ± 2^b^
[*A. marplatensis* + *B. velezensis*] (wheat grains)	208 ± 2^e^	0%	124.3 ± 4^d^	44.8 ± 0.8^bcd^	29.3 ± 2^b^	59 ± 1^cde^	38.8 ± 2^d^	12.7 ± 2^bcd^	26.3 ± 1^c^	37.4 ± 2^d^	17 ± 2^d^
Mean	170	0	106.6	36.3	26.6	50	34.4	11.6	25.4	24.9	15
LSD (0.05)	Leaves no.T:2.093, F: 1.186, TF 2.426—Shoot wet wt. T: 1.041, F: 1.472, TF: 2.082—Shoot dry wt. T: 0.179, F: 0.113, TF: 0.253—Shoot length: T: 0.194, F: 0.123, TF: 0.275—Branches: T: 0.174, F: 0.264. TF: 0.389—Root wet wt. T: 0.078, F: 0.87, TF: 0.155—Root dry wt. T: 0.558, F: 0.353, TF: 0.789—Root length: T: 0.499, F: 0.623, TF: 1.116—Tuber wt. T: 1.041, F: 1.472, TF: 2.082—Tuber No. T: 0.012, F: 0.023, TF: 0.074 The data are the mean of three replicates ± SE Means having the same letter are not significantly different using Duncan’s multiple range test (DMRT) (*P* < 0.05)										

Our results noticed that the optimum temperature and pH for antagonistic activity effect against pathogen *F. oxysporum* was at 35 °C for *A. marplatensis* 53 mm and *B. velezensis* 9 mm at 30 °C after incubation for 24 h. It was noticed that the optimum pH at 6.5 for *A. marplatensis* was 47.2 mm and for *B. velezensis* 8 mm, for which *A. marplatensis* showed the highest antagonistic activity effect. El-Sersawy et al. (2022) reported that, the strains showed efficacy to inhibit the growth of *F. oxysporum* in-vitro with percentages of 56.2, 51.3, and 46.2% for *Achromobacter sp.*, *B. haynesii*, and *B. paramycoides*, respectively at pH 7 and 35 °C for 48 h. Yi et al. (2022) said that, antagonistic strain *B. mojovensis* isolated from wheat roots acts as an antifungal against *Fusarium* sp. and to improve the antifungal activity the culture conditions were at an initial pH of 7.50, and 35 °C for 72 h.

The results indicated that the optimum temperature for the antagonistic activity effect against pathogen *R. solanacearum* was at 35°C for *A. marplatensis* 8 mm and *B. velezensis* 22 mm at 30 °C which showed the highest antagonistic activity effect. The results indicated that, the optimum pH for the antagonistic activity effect against pathogen *R. solanacearum* was at pH 6.5 for *A. marplatensis* 8 mm and *B. velezensis* 29 mm, which showed the highest antagonistic activity effect. Mandal et al. (2017) showed that, the most virulent pathogen *R. solanacearum* was controlled by an indigenous antagonistic soil bacteria *Bacillus cereus* at pH7 and 30 °C. Cao et al. (2018) mentioned that, *B. velezensis* possessing potent antagonistic activity against *R. solanacearum* and *F. oxysporum* were characterized under laboratory and greenhouse conditions. Three lipopolypeptide (LP) compounds have been identified as responsible for the antimicrobial activity of these two strains. Our results indicated that, a gradual decrease in the bacterial count of *A. marplatensis* was shown after being exposed to For *A. marplatensis* and *B. velezensis*, the results indicated that a gradual decrease in the bacterial count was shown after exposure to UV radiation at different times 1h and 2h, while no noticeable growth rate when exposed for 3 h compared to control after incubation for 24–48h.

Bacteria in exponential, stationary, or death phase may be prone to UV damage to different degrees depending on the fidelity and the accuracy of the DNA repair systems. The survival of the cells in response to UV irradiation was assessed. In the stationary phase (Abedi et al. 2004).

The effect of *A. marplatensis* and *B. velezensis* on the *S. tuberosum* shoot which was grown in sandy clay loamy soil was detected after cultivation for 90 days on wheat grains or in a broth media. Adding probiotics together *A. marplatensis* and *B. velezensis* showed the most effective protective results. As a result, using probiotics in broth was better than using wheat grains.

The antagonistic effect with dual inoculation of *A. marplatensis* and *B. velezensis* on *S. tuberosum* plants artificially infected shoot and root with *R. solanacearum* as a pathogen was detected after cultivation for 90 days.

The best effect was observed with probiotic isolates together against *R. solanacearum* in broth exceeded using them separately or with wheat grains, indicating the synergistic effect of both probiotics against *R. solanacearum*. The biocontrol experiment was screened in vivo to simulate natural biology during the cultivation process in vivo to confirm the positive results which obtained in the laboratory. Abdel-Rahman et al. (2017) reported that, the results revealed the importance of soil inoculation with *A. marplatensis* and its role in increasing soil enzymatic activity. Yan et al. (2021) showed that, field trials have shown that *B. velezensis* can be used to reduce food loss caused by late blight, achieving reductions in late blight of 40.79% (2018) and 37.67% (2019) in potatoes.

The addition of probiotics separately either on wheat grains or in broth improved most of the growth parameters over the control. The effect of using both probiotics together against *F. oxysporum* exceeded using them separately, indicating the synergistic effect of both probiotics against *F. oxysporum*. Bafti et al. (2005) reported that, the efficacy of *Achromobacter sp.* introduced into the culture substrate in suppressing or reducing fungal disease was estimated by assessing the progression of wilt disease severity in plants inoculated with *F. oxysporum* compared to untreated control plants. The isolate *Achromobacter sp.* significantly (*P* < 0.05) reduced the disease severity on watermelon plants 20 days after inoculation. Watermelon cotyledons inoculated with *F. oxysporum*, in the absence of *Achromobacter sp.* showed very progressive symptoms of *Fusarium* wilt: brown necrotic spots, vascular discoloration on roots, collar breakage, severe wilting, burns, and death 20 days after inoculation. In the presence of *Achromobacter sp.* no pathological symptoms were observed, increased plant growth, decreased vascular wilt on stems, leaves and roots, and increased length and weight of watermelon roots. The disease severity percentage decreased in infected watermelon plants treated with *Achromobacter sp*. bacterial suspension by 57% compared to untreated inoculated plants. In addition to suppressing *Fusarium* wilt and the incidence of root rot, the bacteria increased plant growth and root system development compared to untreated control plants. Registeri et al. (2012) mentioned that, other antagonistic bacteria were tested to manage *Fusarium* wilt of melon under greenhouse conditions, including *Burkholderia sp., Bacillus sp., Streptomyces sp*. and *Pseudomonas fluorescens*, isolated from rhizospheric soil of melon fields. They showed to be capable of promoting plant growth and reducing vascular wilt disease by producing antibiotic and exogenous cell liquid. (Nawangsih Abdjad et al. 2011) said that, the selected isolates were tested in greenhouse on the antagonistic effect against *R. solanacearum* in culture assays obtained *B. amyloliquefaciens* isolates as promising biocontrol agents. At six weeks after transplanting, plants treated with promising biocontrol agents showed significantly lower disease incidence (33%) than that of control (83%).

## Conclusion

The addition of both probiotic isolates either broth or wheat grains load separately has enhanced all the growth parameters, however, the better results and increased production was in favor of adding probiotics with broth more than wheat. On the other hand, both probiotics showed a remarkable protective effect against potato pathogens separately and reduced the negative impact of the infection using them together.

## Supplementary Information

Below is the link to the electronic supplementary material.

Supplementary file1 (TIF 1715 KB)

Supplementary file2 (TIF 1063 KB)

Supplementary file3 (DOCX 632 KB)

Supplementary file4 (DOCX 555 KB)

Supplementary file5 (DOCX 68 KB)

## Data Availability

The identified strains in this study have been deposited in NCBI database under the accession numbers OQ457665 and OQ457694.
