# Supplementary figures and images for: In vivo application of potent probiotics for enhancing potato growth and controlling Ralstonia solanacearum and Fusarium oxysporum infections
Source: Antonie Van Leeuwenhoek. 2024 Feb 9;117(1):33. doi: 10.1007/s10482-024-01928-2 (PMC10858073; doi:10.1007/s10482-024-01928-2)

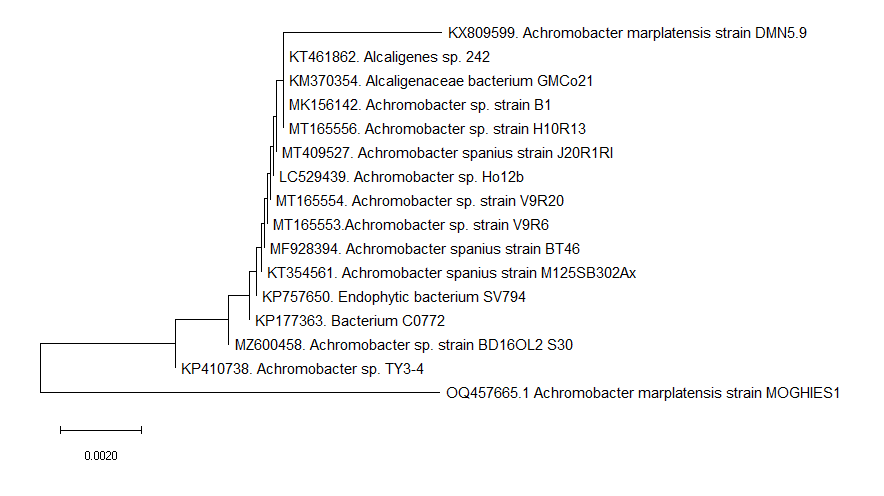

Supplement: Supplementary file 1 — Supplementary file1 (TIF 1715 KB) [file 10482_2024_1928_MOESM1_ESM.tif]

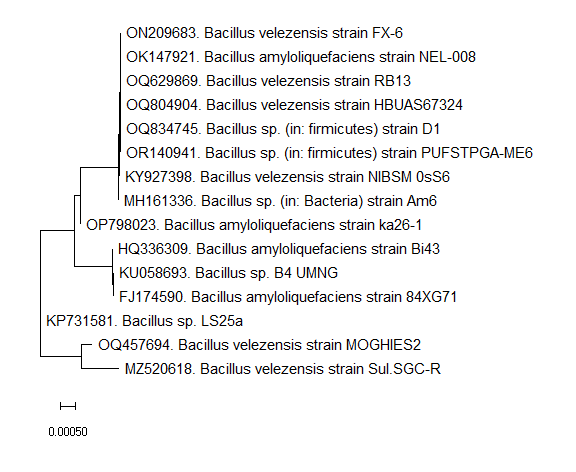

Supplement: Supplementary file 2 — Supplementary file2 (TIF 1063 KB) [file 10482_2024_1928_MOESM2_ESM.tif]
