# Supplementary material for: In vivo application of potent probiotics for enhancing potato growth and controlling Ralstonia solanacearum and Fusarium oxysporum infections
Source: Antonie Van Leeuwenhoek. 2024 Feb 9;117(1):33. doi: 10.1007/s10482-024-01928-2 (PMC10858073; doi:10.1007/s10482-024-01928-2)
Supplement: Supplementary file 3 — Supplementary file3 (DOCX 632 KB) [file 10482_2024_1928_MOESM3_ESM.docx]

**
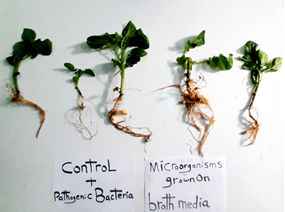
**

**
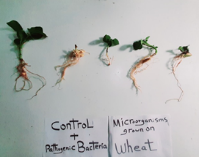
Figure (36) Potato plants inoculated with pathogenic bacteria *R. solancerium* loaded on broth media.**

**Figure (37) Potato plants inoculated with pathogenic bacteria *R. solancerium* loaded on wheat grains.**

**
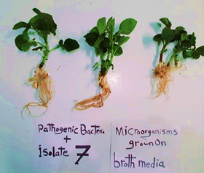
**

**
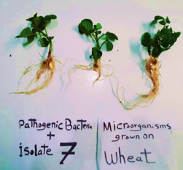
Figure (38) Potato plants inoculation with [*A*. *marplatensis* + *R. solancerium*] loaded on broth media.**

**Figure (39) Potato plants inoculation with [*A*. *marplatensis* + *R. solancerium*] loaded on wheat grains.**

**
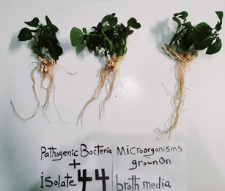
**

**
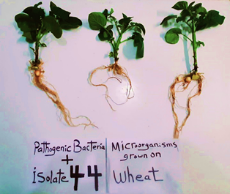
Figure (40) Potato plants inoculated with [*B*. *velezensis* + *R. solancerium*] loaded on broth media.**

**
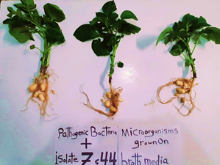
Figure (41) Potato plants inoculated with [*B*. *velezensis* + *R. solancerium*] loaded on wheat grains.**

**
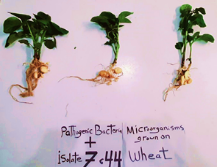
Figure (42) Potato plants inoculated with [*A*. *marplatensis* + *B*. *velezensis* + *R.solancerium*] loaded on broth media.**

**Figure (43) Plants inoculation with [*A*. *marplatensis* + *B*. *velezensis* + *R. solancerium*] loaded on wheat grains.**
