# Supplementary material for: In vivo application of potent probiotics for enhancing potato growth and controlling Ralstonia solanacearum and Fusarium oxysporum infections
Source: Antonie Van Leeuwenhoek. 2024 Feb 9;117(1):33. doi: 10.1007/s10482-024-01928-2 (PMC10858073; doi:10.1007/s10482-024-01928-2)
Supplement: Supplementary file 4 — Supplementary file4 (DOCX 555 KB) [file 10482_2024_1928_MOESM4_ESM.docx]

**
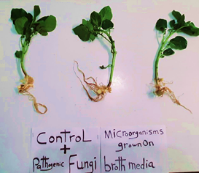
**

**
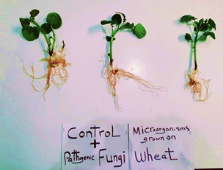
Figure (44) Potato plants inoculated with pathogenic Fungi (F. *oxysporum*) loaded on broth media.**

**
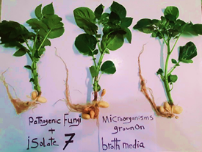
Figure (45) Potato plants inoculated with pathogenic Fungi (F. *oxysporum*) loaded on wheat grains.**

**
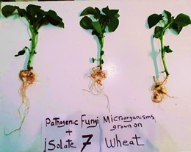
Figure (46) Potato plants inoculated with [A. *marplatensis* + F. *oxysporum*] loaded on broth media.**

**Figure (47) Potato plants inoculated with [A. *marplatensis* + F. *oxysporum*] loaded on wheat grains.**

**
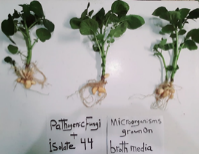
**

**
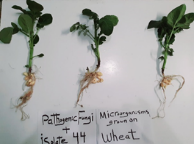
Figure (48) Potato plants inoculated with [B. *velezensis* + F. *oxysporum*] loaded on broth media.**

**
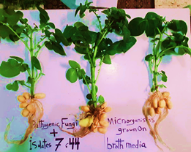
Figure (49) Potato plants inoculated with [B. *velezensis* + F. *oxysporum*] loaded on wheat grains.**

**Figure (50) Potato plants inoculated with [A. *marplatensis* + B. *velezensis* +F. *oxysporum*] loaded on broth media.**

**
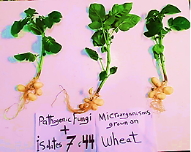
**

**Figure (51) Potato plants inoculated with [A. *marplatensis* + B. *velezensis* +F. *oxysporum*] loaded on wheat grains.**
