# Supplementary material for: In vivo application of potent probiotics for enhancing potato growth and controlling Ralstonia solanacearum and Fusarium oxysporum infections
Source: Antonie Van Leeuwenhoek. 2024 Feb 9;117(1):33. doi: 10.1007/s10482-024-01928-2 (PMC10858073; doi:10.1007/s10482-024-01928-2)
Supplement: Supplementary file 5 — Supplementary file5 (DOCX 68 KB) [file 10482_2024_1928_MOESM5_ESM.docx]

**4.5. Performance.**

The purpose was to determine the optimal conditions for the production of IAA and cellulase, and the biological resistance of pathogenic organisms, such as *F. Oxysporum* and *R. solancerium*, by determining the optimal temperature and pH for the growth of the selected endophytic bacterial isolates, which were defined as A. *marplatensis* and *B. velezensis*.

**4.5.1. Performance of IAA production.**

The activity of production of IAA was determined. The optimum parameters for IAA production from the efficient isolates *A. marplatensis* and *B. velezensis* were determined and recorded. After incubation at different temperatures of 25, 30, 35, and 40°C, it was noticed that the optimum temperature for *A. marplatensis* was observed at 35°C whereby significantly the maximum yield of 66.3, and 61.6 ug/ml was observed at 30 °C by *B. velezensis* as shown in (table 16) (figure 12) . The optimum pH for *A. marplatensis* was observed at pH 6.5, whereby the maximum yield was 66.1, and 62.7 ug/ml was observed at pH 7.5, as shown in (table 17) (figure 13).

**Table (16) Effect of temperature on production of Indole Acetic Acid ug/ml.**

| **Temp / Bacteria** | ***Achromobacter marplatensis*** | ***Bacillus velezensis*** |
| --- | --- | --- |
| **25 ^o^C** | 37.2 ±2.3^de^ | 32.5 ± 1.7^a^ |
| **30 ^o^C** | 50.1 ± 3.1^cde^ | 61.6 ± 2.1^a^ |
| **35 ^o^C** | 66.3 ± 3.4^ab^ | 53.2 ± 1.8^b^ |
| **40 ^o^C** | 47.1 ± 2.5^e^ | 40.9 ± 1.2^b^ |
| **Mean** | 50.2 | 47 |
| **LSD (0.05)** | **T: 0.019 F: 0.030 TF: 0.017** | |

The data are the mean of three replicates ± SE

Means having the same letter are not significantly different using Duncan’s multiple range test (DMRT) (P < 0.05).

**Table (17) Effect of pH on production of Indole Acetic Acid (IAA) µg/ml.**

| **pH / Bacteria** | ***Achromobacter marplatensis*** | ***Bacillus velezensis*** |
| --- | --- | --- |
| **5.5** | 35.5 ±1.2^a^ | 33.1 ±0.9^c^ |
| **6.5** | **66.1 ±2.4^abc^** | 54.7±1.8^d^ |
| **7.5** | 58.9 ±1.2^bcd^ | **62.7±1.1^d^** |
| **Mean** |  |  |
| **LSD (0.05)** | **T: 0.017 F: 0.029 TF: 0.051** | |

The data are the mean of three replicates ± SE

Means having the same letter are not significantly different using Duncan’s multiple range test (DMRT) (P < 0.05).

**4.5.2. Performance of cellulase production**

The optimum parameters for cellulase production from the probiotics *A. marplatensis* and *B. velezensis* were determined and recorded. The optimum temperature for cellulase activity was 35°C for *A. marplatensis* 3.90 *mm* and *B. velezensis* 2.6 *mm* at 30°C, which showed the highest enzyme activity (table 18) (figure 14). It was noticed that the optimum pH of 6.5 for *A. marplatensis* and *B. velezensis* significantly was observed at pH 7.5 whereby maximum yield 3.50 and 2.86 *mm* respectively as shown in (table 19) (figure 15).

**Table (18) Effect of temperature on production of cellulase enzyme activity [Zone/colony (mm)].**

| **Temp / Bacteria** | ***Achromobacter marplatensis*** | ***Bacillus velezensis*** |
| --- | --- | --- |
| **25 ^o^C** | 1.26 ±0.2^de^ | 1.50 ±0.3^c^ |
| **30 ^o^C** | 3.48 ±0.1^c^ | **2.6 ±0.3^a^** |
| **35 ^o^C** | **3.90 ±0.1^de^** | 2.46 ±0.2^cd^ |
| **40 ^o^C** | 1.48 ±0.2^bc^ | 1.98 ±0.1^bc^ |
| **Mean** |  |  |
| **LSD (0.05)** | **T: 0.011 F: 0.021 TF: 0.027** | |

The data are the mean of three replicates ± SE

Means having the same letter are not significantly different using Duncan’s multiple range test (DMRT) (P < 0.05).

**Table (19) Effect of pH on production of cellulase enzyme [Zone/colony (mm)].**

| **pH / Bacteria** | ***Achromobacter marplatensis*** | ***Bacillus velezensis*** |
| --- | --- | --- |
| **5.5** | 1.73 ± 0.2^ab^ | 0.76 ± 0.1^h^ |
| **6.5** | **3.50** ± 0.5^c^ | 1.62 ± 0.2^cd^ |
| **7.5** | 2.25± 0.4^bc^ | **2.86** ± 0.3^c^ |
| **Mean** |  |  |
| **LSD (0.05)** | **T: 0.08 F: 0.17 TF: 0.19** | |

The data are the mean of three replicates ± SE

Means having the same letter are not significantly different using Duncan’s multiple range test (DMRT) (P < 0.05).

**4.5.3. Effect of antagonistic activity between probiotics and against *F. oxysporum***

The optimum temperature for antagonistic activity against *F. oxysporum* was 35°C for *A. marplatensis* 53 *mm* and *B. velezensis* 9 *mm* at 30°C, and *A. marplatensis* showed the highest antagonistic activity after incubation for 24 h (table 20) (figure 16). The optimum pH for the antagonistic activity against *F. oxysporum* significantly was pH 6.5 for *A. marplatensis* 47.2 *mm* and *B. velezensis* 8 *mm*, where *A. marplatensis* showed the highest antagonistic activity effect, as shown in (table 21) (figure 17).

**Table (20) Effect of temperature on antagonistic activity effect against *Fusarium oxysporum.***

| **Temperature / bacteria** | ***Achromobacter marplatensis*** | ***Bacillus velezensis*** |
| --- | --- | --- |
| **25 ^o^C** | 19 mm ± 1.4^c^ | 7 mm ± 0.11^bc^ |
| **30 ^o^C** | 45 mm ± 2.8^a^ | **9 mm ± 0.15^de^** |
| **35 ^o^C** | **53 mm ± 2.8^cd^** | 8 mm ± 0.14^c^ |
| **40 ^o^C** | 33 mm ± 1.6^bc^ | 8 mm ± 0.11^a^ |
| **Mean** |  |  |
| **LSD (0.05)** | **T: 0.020 F: 0.032 TF: 0.042** | |

The data are the mean of three replicates ± SE

Means having the same letter are not significantly different using Duncan’s multiple range test (DMRT) (P < 0.05).

**Table (21) Effect of pH on antagonistic activity effect against *Fusarium oxysporum (mm).***

| **pH / Bacteria** | ***Achromobacter marplatensis*** | ***Bacillus velezensis*** |
| --- | --- | --- |
| **5.5** | 29 ± 3.0^b^ | 7 ± 0.12^a^ |
| **6.5** | **47.2 ±3.5^ef^** | 7 ± 0.19^cd^ |
| **7.5** | 38.4 ± 2.3^i^ | **8 ± 0.15^hi^** |
| **Mean** |  |  |
| **LSD (0.05)** | **T: 0.014 F: 0.022 TF: 0.034** | |

The data are the mean of three replicates ± SE

Means having the same letter are not significantly different using Duncan’s multiple range test (DMRT) (P < 0.05).

**4.5.4. Effect of antagonistic activity between probiotics and *R. solanacearum*.**

The results indicated that the optimum temperature for the antagonistic activity effect against pathogen *R. solanacearum* was at 35°C for *A. marplatensis* 8 *mm* and *B. velezensis* 22 *mm* at 30 °C, which showed the highest antagonistic activity effect, as shown in (table 22) (figure 18). The results indicated that the optimum pH for antagonistic activity against *R. solanacearum* was pH 6.5 for *A. marplatensis* 8 *mm* and *B. velezensis* 29 *mm*, which showed the highest antagonistic activity effect, as shown in (table 23) (figure 19).

**Table (22) Effect of temperature on antagonistic activity effect against *Ralstonia solanacearum (mm).***

| **Temp / Bacteria** | ***Achromobacter marplatensis*** | ***Bacillus velezensis*** |
| --- | --- | --- |
| **25 ^o^C** | 6 ± 0.15^b^ | 8 ± 0.9^b^ |
| **30 ^o^C** | 7 ± 0.15^bc^ | **22 ± 1.3^de^** |
| **35 ^o^C** | **8 ± 0.15^ghi^** | 16 ± 1.2^de^ |
| **40 ^o^C** | 6 ± 0.14^fgh^ | 13 ± 0.9^cd^ |
| **Mean** |  |  |
| **LSD (0.05)** | **T: 0.016 F: 0.040 TF: 0.025** | |

The data are the mean of three replicates ± SE

Means having the same letter are not significantly different using Duncan’s multiple range test (DMRT) (P < 0.05).

**Table (23) Effect of pH on antagonistic activity effect against *Ralstonia solanacearum (mm).***

| **pH / Bacteria** | ***Achromobacter marplatensis*** | ***Bacillus velezensis*** |
| --- | --- | --- |
| **5.5** | 6 ± 0.8^b^ | 17 ± 2.1^b^ |
| **6.5** | **8 ± 0.8^de^** | 24 ± 2.3^de^ |
| **7.5** | 7 ± 0.6^cd^ | **29 ± 2.3^cd^** |
| **Mean** |  |  |
| **LSD (0.05)** | **T: 0.019 F: 0.027 TF: 0.045** | |

The data are the mean of three replicates ± SE

Means having the same letter are not significantly different using Duncan’s multiple range test (DMRT) (P < 0.05).
